# Supplementary material for: The Evolution and Characterization of the RNA Interference Pathways in Lophotrochozoa
Source: Genome Biol Evol. 2024 May 7;16(5):evae098. doi: 10.1093/gbe/evae098 (PMC11114477; doi:10.1093/gbe/evae098)
Supplement: evae098_Supplementary_Data [file evae098_supplementary_data.zip › Supplementary_materials.zip!/selected_miRNAs/NoGe_selected_miRNAs/mirdeep_pdf_files/NMRB01000024.1_Notospermus_geniculatus_strain_Ushimado_scaffold24,_whole_genome_shotgun_sequence_1013.pdf]

Provisional ID	:	NMRB01000024.1_Notospermus_geniculatus_strain_Ushimado_scaffold24,_whole_genome_shotgun_sequence_1013
Score total	:	3873.8
Score for star read(s)	:	3.9
Score for read counts	:	3865.6
Score for mfe	:	1.2
Score for randfold	:	0
Score for cons. seed	:	3
Total read count	:	7594
Mature read count	:	7501
Loop read count	:	0
Star read count	:	93

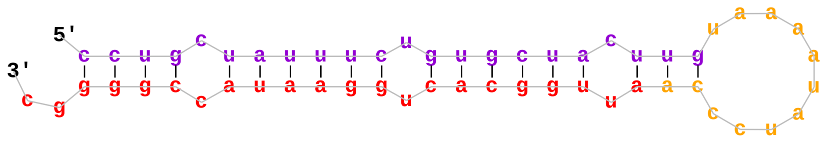

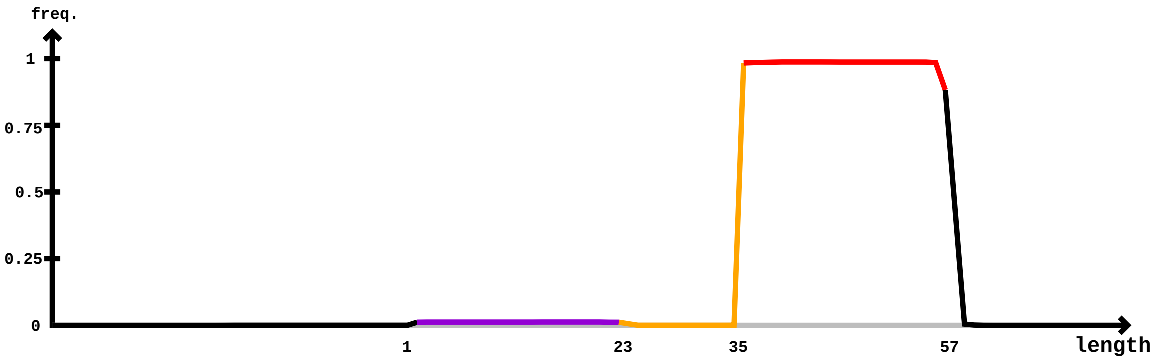

Star **Mature**

```
5'- aggaaaagaauucauagaagcuaucuuuuggaaugcccugcuauuucugugcuacuuguaaaauaucccaauuggcacuggaauaccggggcauuuuccuggaauguacc   -3'   obs
    aggaaaagaauucauagaagcuaucuuuuggaaugcccugcuauuucugugcuacuuguaaaauaucccaauuggcacuggaauaccggggcauuuuccuggaauguacc         exp
    (((((((..((((.((((((....))))))))))((((((.((((((.((((((.(((..........))).)))))).)))))).)))))).)))))))..........   reads   mm   sample
    .................aagcuaucuuuuggaaugc..........................................................................   1   0   Mm2
    ....................................ccugcuauuucugugcuacu......................................................   1   0   Mm2
    ....................................ccugcuauuucugugcuacuu.....................................................   1   0   Mm2
    ....................................ccugcuauuucugugcuacuGg....................................................   1   1   Mm2
    ....................................ccugcuauuucugugcuGcuug....................................................   1   1   Mm2
    ....................................ccugcuauuucugugcuacuug....................................................   18   0   Mm2
    .................................................ugcuacuuguaaaauaucccaauuggcacu...............................   1   0   Mm2
    ..........................................................uaaaauaucccaauuggcacug..............................   1   0   Mm2
    .....................................................................Cauuggcacuggaauaccgggc...................   1   1   Mm2
    ......................................................................auuggcacuggaauaccgg.....................   1   0   Mm2
    ......................................................................auuggcacuggaauaccggg....................   7   0   Mm2
    ......................................................................auuggcacuggaauacAgggg...................   2   1   Mm2
    ......................................................................auuggcacuggaauaccAggg...................   3   1   Mm2
    ......................................................................auuggcacuggaauaccgUgg...................   8   1   Mm2
    ......................................................................auuggcacuggaauaccUggg...................   2   1   Mm2
    ......................................................................auuggcacuggaauaccgggg...................   274   0   Mm2
    ......................................................................auuggcacuggaauacUgggg...................   1   1   Mm2
    ......................................................................auuggcacuggaauaccCggg...................   1   1   Mm2
    ......................................................................auuggcacuggaauaccgggC...................   1   1   Mm2
    ......................................................................Uuuggcacuggaauaccgggg...................   1   1   Mm2
    ......................................................................auuggcacugCaauaccgggg...................   1   1   Mm2
    ......................................................................auuggcacuggaauacGggggc..................   4   1   Mm2
    ......................................................................auuggcacugAaauaccggggc..................   3   1   Mm2
    ......................................................................auuggcacuggaauaUcggggc..................   1   1   Mm2
    ......................................................................auuggcacuggaauaccUgggc..................   15   1   Mm2
    ......................................................................auuggcacuggaauaccggggc..................   2683   0   Mm2
    ......................................................................auuggcacAggaauaccggggc..................   1   1   Mm2
    ......................................................................auugCcacuggaauaccggggc..................   1   1   Mm2
    ......................................................................auuggcacuggaauaccgCggc..................   1   1   Mm2
    ......................................................................auuggcacuggaauaccggCgc..................   1   1   Mm2
    ......................................................................auuggcacuggaauaccAgggc..................   18   1   Mm2
    ......................................................................auuggGacuggaauaccggggc..................   1   1   Mm2
    ......................................................................auuggcacuggaauaccggggG..................   1   1   Mm2
```

```
                                    Star                                    Mature
aggaaaagaauucauagaagcuaucuuuuggaaugcccugcuauuucugugcuacuuguaaaauauccccaauuggcacuggaauaccggggcauuuuccuggaauguacc
......................................................................auuAgcacuggaauaccggggc..................   1      1   Mm2
......................................................................auuggcacuggaauUccggggc..................   2      1   Mm2
......................................................................auCggcacuggaauaccggggc..................   1      1   Mm2
......................................................................auuggcacugUaauaccggggc..................   2      1   Mm2
......................................................................auuggcacuggaauaGcggggc..................   1      1   Mm2
......................................................................auuggcacugCaauaccggggc..................   2      1   Mm2
......................................................................auuggcacuggaaCaccggggc..................   1      1   Mm2
......................................................................auGggcacuggaauaccggggc..................   1      1   Mm2
......................................................................auuggcacuggaauacUggggc..................   5      1   Mm2
......................................................................Uuuggcacuggaauaccggggc..................   5      1   Mm2
......................................................................auuggcacuUgaauaccggggc..................   1      1   Mm2
......................................................................auuggcacCggaauaccggggc..................   1      1   Mm2
......................................................................auuggcacuggaauaccggAgc..................   1      1   Mm2
......................................................................auuggcacuggaauaccgggAc..................   1      1   Mm2
......................................................................auuggcacuggaauaccCgggc..................   14     1   Mm2
......................................................................auuggcacuggaauaccggggU..................   21     1   Mm2
......................................................................aAuggcacuggaauaccggggc..................   1      1   Mm2
......................................................................auuggcacuggaauaccggUgc..................   1      1   Mm2
......................................................................auuggcacuggaauaccggggA..................   1      1   Mm2
......................................................................auuggcUcuggaauaccggggc..................   1      1   Mm2
......................................................................Nuuggcacuggaauaccggggc..................   1      1   Mm2
......................................................................Cuuggcacuggaauaccggggc..................   1      1   Mm2
......................................................................auuggcacuggGauaccggggc..................   3      1   Mm2
......................................................................auuggcacuggaauacAggggc..................   11     1   Mm2
......................................................................auuggcacuggaauaccggggcU.................   19     1   Mm2
......................................................................auuggcacuggaauaccggggAa.................   2      1   Mm2
......................................................................auuggcacuggaauaccggggca.................   3      0   Mm2
......................................................................auuggcacuggaauaccggggcUu................   4      1   Mm2
......................................................................auuggcacuggaauaccggggcau................   2      0   Mm2
......................................................................auuggcacuggaauaccggggcUuu...............   1      1   Mm2
......................................................................auuggcacuggaauaccggggcUuuu..............   1      1   Mm2
.......................................................................uuggcacuggaauaccgggg...................   1      0   Mm2
.......................................................................uuggcacuggaauaccggggca.................   3      0   Mm2
........................................................................uggcacuggaauaccggggc..................   4      0   Mm2
.........................................................................ggcacuggaauaccggggc..................   4      0   Mm2
..........................................................................gcacuggaauGccggggc..................   1      1   Mm2
..........................................................................gcacuggaauaccggggc..................   2      0   Mm2

....................................ccugcuauuucugugcuCcuug....................................................   1      1   Mm1
....................................ccugcuauuuAugugcuacuug....................................................   1      1   Mm1
....................................ccugcuauuucugugcuacuug....................................................   14     0   Mm1
......................................................................Nuuggcacuggaauaccgggg...................   1      1   Mm1
......................................................................auuggcacuggaauaccAggg...................   1      1   Mm1
......................................................................auuggcacuggaauaccgUgg...................   1      1   Mm1
......................................................................auAggcacuggaauaccgggg...................   1      1   Mm1
......................................................................auuggcacuggaauaccCggg...................   1      1   Mm1
......................................................................auuggcacuggaauaccUggg...................   1      1   Mm1
......................................................................auuggcacuggaauaccgggg...................   181    0   Mm1
......................................................................auuggcacuggaauaccCgggc..................   4      1   Mm1
......................................................................auuggcacuggaauaccggggc..................   1164   0   Mm1
......................................................................auuggcacuUgaauaccggggc..................   1      1   Mm1
......................................................................auuggcacuggaauaccAgggc..................   7      1   Mm1
......................................................................auuggcacuggaauaccUgggc..................   8      1   Mm1
......................................................................auuggcacuggaauaccggggU..................   16     1   Mm1
......................................................................auuggcacuggaauacUggggc..................   1      1   Mm1
......................................................................aAuggcacuggaauaccggggc..................   1      1   Mm1
......................................................................auuggcacuggaauacGggggc..................   1      1   Mm1
......................................................................auuggcacugAaauaccggggc..................   1      1   Mm1
......................................................................auuggcacuggaaCaccggggc..................   1      1   Mm1
......................................................................auuggcacuggaauaccggAgc..................   1      1   Mm1
......................................................................auuggcacuggaaAaccggggc..................   1      1   Mm1
......................................................................auuggcacugCaauaccggggc..................   4      1   Mm1
......................................................................auuggcacuggaauacAggggc..................   8      1   Mm1
......................................................................auuggcacuggaauaccCgggca.................   1      1   Mm1
......................................................................auuggcacuggaauaccggggca.................   6      0   Mm1
......................................................................auuggcacuggaauaccggggcU.................   5      1   Mm1
......................................................................auuggcacuggaauaccggggcaA................   4      1   Mm1
.......................................................................uuggcacuggaauaccggggc..................   3      0   Mm1
.........................................................................ggcacuggaauaccggggc..................   1      0   Mm1

....................................ccugcuauuucugugcuacuuU....................................................   5      1   Fm1
```

```
                                     Star                           Mature
aggaaaagaauucauagaagcuaucuuuuggaaugcccugcuauuucugugcuacuuguaaaauauccccaauuggcacuggaauaccggggcauuuuccuggaauguacc
....................................ccugcuauuucugugcuacuug.....................................................  5     0   Fm1
.....................................cugcuauuucugugcuacuugu....................................................  4     0   Fm1
.......................................................................auuggcacuggaauaccggg....................  1     0   Fm1
.......................................................................auuggcacuggaauaccgUgg...................  4     1   Fm1
.......................................................................auuggcacuggaauacAgggg...................  1     1   Fm1
.......................................................................auuggcacuggaauaccCggg...................  1     1   Fm1
.......................................................................auuCgcacuggaauaccgggg...................  1     1   Fm1
.......................................................................auuggcacuggaauaccgggg...................  73    0   Fm1
.......................................................................auuggcacuggaauaccUgggc..................  7     1   Fm1
.......................................................................Nuuggcacuggaauaccggggc..................  2     1   Fm1
.......................................................................auCggcacuggaauaccggggc..................  2     1   Fm1
.......................................................................auuggcacugCaauaccggggc..................  2     1   Fm1
.......................................................................auuggcacuggaauaccggAgc..................  1     1   Fm1
.......................................................................auuggcacuggaauaccggUgc..................  1     1   Fm1
.......................................................................auuggcacugAaauaccggggc..................  2     1   Fm1
.......................................................................auuggcacugUaauaccggggc..................  3     1   Fm1
.......................................................................auuggcacuggaauacAggggc..................  9     1   Fm1
.......................................................................auuggcacuggaauaccAgggc..................  9     1   Fm1
.......................................................................aAuggcacuggaauaccggggc..................  1     1   Fm1
.......................................................................auuggcacuggaGuaccggggc..................  1     1   Fm1
.......................................................................auuggcacuggaauacUggggc..................  3     1   Fm1
.......................................................................auuggcacuggaauaccCgggc..................  5     1   Fm1
.......................................................................auuggcacuggaauacGggggc..................  2     1   Fm1
.......................................................................Uuuggcacuggaauaccggggc..................  1     1   Fm1
.......................................................................auuggcacuggaauaccggggA..................  6     1   Fm1
.......................................................................auuggcacuggaauaUcggggc..................  1     1   Fm1
.......................................................................auuggcacuggaauaccggggc..................  1741  0   Fm1
.......................................................................auuggcacuggaauaccggggU..................  16    1   Fm1
.......................................................................auuUgcacuggaauaccggggc..................  1     1   Fm1
.......................................................................auuggcacuggaauaccggggG..................  1     1   Fm1
.......................................................................auuggcacuggaauaAcggggc..................  1     1   Fm1
.......................................................................auuggcacuUgaauaccggggc..................  1     1   Fm1
.......................................................................auuggcacuggaauaccgUggc..................  1     1   Fm1
.......................................................................auuggNacuggaauaccggggc..................  1     1   Fm1
.......................................................................auuggcacuggaauaccggggcU.................  37    1   Fm1
.......................................................................auuggcacuggaauaccggggca.................  7     0   Fm1
.......................................................................auuggcacuggaauaccggggUau................  3     1   Fm1
.......................................................................auuggcacuggaauaccggggcUu................  12    1   Fm1
.......................................................................auuggcacuggaauaccggggcUuu...............  5     1   Fm1
.......................................................................auuggcacuggaauaccggggcaGu...............  1     1   Fm1
...........................................................................gcacuggaauaccggggc..................  1     0   Fm1

....................................ccugcuauuucugugcuacuug.....................................................  25    0   Fm2
.......................................................................Nuuggcacuggaauaccgggg...................  1     1   Fm2
.......................................................................auuggcacuggaauaccgggg...................  53    0   Fm2
.......................................................................auuggcacuggaauaccgUgg...................  3     1   Fm2
.......................................................................auuggcacuggaauaccCggg...................  2     1   Fm2
.......................................................................auuggcacuggaauaccUggg...................  1     1   Fm2
.......................................................................auuggcacuggaauacUggggc..................  2     1   Fm2
.......................................................................auuggcacuggaauaccggggU..................  3     1   Fm2
.......................................................................Uuuggcacuggaauaccggggc..................  1     1   Fm2
.......................................................................auuggcacuggaauaccggggc..................  146   0   Fm2
.......................................................................auuggcacuggaauaccCgggc..................  1     1   Fm2
.......................................................................auuggcacuggaauaccAgggc..................  1     1   Fm2
.......................................................................auuggcacuggaauaccggggca.................  3     0   Fm2
........................................................................uuggcacuggaauaccgggg...................  2     0   Fm2
........................................................................uuggcacuggaauaccggggc..................  1     0   Fm2

................gaagcuaucuuuuggaaugc...........................................................................  1     0   Mm3
....................................ccugcuauuucugugcuacuug.....................................................  11    0   Mm3
.......................................................................auuggcacuggaauaccggg....................  3     0   Mm3
.......................................................................auuggcacuggaauaccAggg...................  2     1   Mm3
.......................................................................auuggcacuggaauaccUggg...................  2     1   Mm3
.......................................................................auuggcacuggaauaccgUgg...................  2     1   Mm3
.......................................................................auuggcacuggaauaccgggg...................  84    0   Mm3
.......................................................................auuggcacuggaauaccCggg...................  1     1   Mm3
.......................................................................auuggcacuggaauacAggggc..................  2     1   Mm3
.......................................................................auuggcacugUaauaccggggc..................  1     1   Mm3
.......................................................................auuggcacuggaauaccgCggc..................  1     1   Mm3
.......................................................................auuggcacugAaauaccggggc..................  2     1   Mm3
```

Star **Mature**

```
aggaaaagaauucauagaagcuaucuuuuggaaugcccugcuauuucugugcuacuuguaaaaauaucccaauuggcacuggaauaccggggcauuuuccuggaauguacc
.......................................................................aCuggcacuggaauaccggggc..................   1     1   Mm3
.......................................................................auuAgcacuggaauaccggggc..................   1     1   Mm3
.......................................................................auuggcacuggaauacUggggc..................   1     1   Mm3
.......................................................................auuggcacuggaauaccggggc..................   441   0   Mm3
.......................................................................auuggcacuggaauaccUgggc..................   4     1   Mm3
.......................................................................auuggcacuggaauaccgggCc..................   1     1   Mm3
.......................................................................auuggcacuggaauaccggggU..................   6     1   Mm3
.......................................................................auuggAacuggaauaccggggc..................   1     1   Mm3
.......................................................................auuggcacuggaauaccgggUc..................   1     1   Mm3
.......................................................................auuggcacuggaauaccCgggc..................   3     1   Mm3
.......................................................................Uuuggcacuggaauaccggggc..................   1     1   Mm3
.......................................................................auuggcacuggaauaccAgggc..................   3     1   Mm3
.......................................................................auuggcacuggaauaccggggAa.................   1     1   Mm3
.......................................................................auuggcacuggaauaccggggcU.................   7     1   Mm3
.......................................................................auuggcacuggaauaccggggca.................   5     0   Mm3
.......................................................................auuggcacuggaauaccggggcUu................   1     1   Mm3
.......................................................................auuggcacuggaauaccggggcGu................   1     1   Mm3
........................................................................uuggcacuggaauaccggggc..................   1     0   Mm3
.........................................................................uggcacuggaauaccggggc..................   1     0   Mm3
..........................................................................ggcacuggaauaccggggc..................   3     0   Mm3
...........................................................................Acacuggaauaccggggc..................   1     1   Mm3

....................................ccugcuauuucugugcuacu.......................................................   4     0   Fm3
....................................ccugcuauuucugugcuacuug.....................................................   1     0   Fm3
.......................................................................auuggcacuggaauaccggg....................   5     0   Fm3
.......................................................................auuggcacuggaauaccgggg...................   58    0   Fm3
.......................................................................auuggcacuggaauaccgggA...................   1     1   Fm3
.......................................................................auuggcacugAaauaccgggg...................   1     1   Fm3
.......................................................................auuggcacuggaauacAgggg...................   1     1   Fm3
.......................................................................auuggcacuggaauaccggAg...................   1     1   Fm3
.......................................................................auuggcacuggaauaccggggc..................   69    0   Fm3
.......................................................................auuggcacuggaauaccggAgc..................   1     1   Fm3
.......................................................................auuggcacuggaauaccggggU..................   2     1   Fm3
.......................................................................auuggcacuggaauacUggggc..................   1     1   Fm3
.......................................................................auuggcacuggaauaccggggca.................   1     0   Fm3
.......................................................................auuggcacuggaauaccggggcau................   1     0   Fm3
```
